# Polarization of the endomembrane system is an early event in fucoid zygote development

**DOI:** 10.1186/1471-2229-6-5

**Published:** 2006-02-23

**Authors:** Rhett Hadley, Whitney E Hable, Darryl L Kropf

**Affiliations:** 1 University of Utah, Department of Biology, 257 South 1400 East, Salt Lake City, Utah 84112-0840, USA; 2 Department of Biology, University of Massachusetts Dartmouth, 285 Old Westport Road, North Dartmouth MA 02747, USA

## Abstract

**Background:**

Fucoid zygotes are excellent experimental organisms for investigating mechanisms that establish cell polarity and determine the site of tip growth. A common feature of polarity establishment is targeting endocytosis and exocytosis (secretion) to localized cortical domains. We have investigated the spatiotemporal development of endomembrane asymmetry in photopolarizing zygotes, and examined the underlying cellular physiology.

**Results:**

The vital dye FM4-64 was used to visualize endomembranes. The endomembrane system preferentially accumulated at the rhizoid (growth) pole within 4 h of fertilization. The polarized endomembrane array was initially labile and reoriented when the developmental axis changed direction in response to changing light cues. Pharmacological studies indicated that vesicle trafficking, actin and microtubules were needed to maintain endomembrane polarity. In addition, endocytosis required a functional cortical actin cytoskeleton.

**Conclusion:**

Endomembrane polarization is an early event in polarity establishment, beginning very soon after photolocalization of cortical actin to the presumptive rhizoid site. Targeting of endocytosis and secretion to the rhizoid cortex contributes to membrane asymmetry. We suggest that microtubule-actin interactions, possibly involving microtubule capture and stabilization at actin-rich sites in the rhizoid, may organize the endomembrane array.

## Background

Establishing cell polarity in eukaryotes is dependent upon highly regulated endomembrane cycling (endocytosis, exocytosis and inter-organelle trafficking) [1-3]. Regulated endomembrane cycling allows for delivery and removal of specific molecules at localized sites, which facilitates formation of morphogenic gradients and assembly of unique cortical domains that control growth, division and development [4-6]. Tip growth represents an extreme form of cell polarity and is distributed throughout the eukaryotic domain. In many tip-growing cells, including fission and budding yeast, fungal hyphae, algal rhizoids, root hairs and pollen tubes, the endomembrane system is focused toward the elongating apex [7-9]. A polarized endomembrane system not only allows local deposition of membrane and wall precursors needed for sustained cell elongation, but also facilitates formation of distinct apical and subapical domains in the tip [4,10]. Although much less is known about the mechanisms that establish and maintain a polar endomembrane system in tip-growing cells, the actin cytoskeleton appears to be intimately involved. Actin is thought to serve several functions in endomembrane polarization including organizing the endomembrane array, creating a target site for exocytosis, transporting vesicles and spatially restricting the site of endocytosis [1,3,5,10].

We are interested in the processes that establish polarity in tip-growing cells and this study focuses on formation and maintenance of a polarized endomembrane array in *Silvetia *zygotes. Zygotes of fucoid algae, genera *Fucus *and *Silvetia*, provide an excellent paradigm for investigating cell polarity establishment [5,6,11-13]. Symmetry in the egg is broken at fertilization and the sperm entry site defines the rhizoid pole of a growth axis [14]. This initial axis can be overridden by vectorial cues in the environment, such as unilateral light, which induces a new rhizoid pole on the shaded hemisphere. The rhizoid pole is marked by a cortical patch of dynamic actin that repositions rapidly by depolymerization/repolymerization during axis realignment [15,16]. Beginning about 4 h after fertilization (AF), the existing axis becomes amplified by targeting secretion to the actin patch at the rhizoid pole [8] and by formation of cytosolic H^+ ^[17] and Ca^2+ ^[18] gradients. Surprisingly, the amplified axis remains labile for several hours and becomes fixed only an hour or so prior to germination (12 h AF), when growth becomes visibly localized to the rhizoid pole.

Endomembranes clearly accumulate preferentially in the rhizoid tip of germinated zygotes [19,20]; however, the timing of endomembrane polarization remains a major unresolved issue. The occurrence of asymmetric adhesive secretion by 4 h AF [8] is consistent with polarization of the endomembrane system toward the rhizoid pole at this early amplification stage. However, a previous study found that the endomembrane system remains uniformly distributed until germination [20], implying that polar secretion ensues well before endomembrane polarization. To resolve the spatiotemporal progression of endomembrane polarization, we have used a fluorescent membrane probe, FM4-64, to visualize endomembrane arrays in *Silvetia compressa *zygotes. FM4-64 is an amphiphilic styryl dye that has proved quite useful for investigating endocytosis and membrane trafficking, including trafficking in tip-growing cells [20-23]. The dye inserts into the outer leaflet of the plasma membrane and is internalized by endocytosis [2,21,24]. It fluoresces weakly in an aqueous environment but fluorescence increases dramatically when the dye inserts into membranes [24]. Following endocytosis, the dye remains in membranes and is redistributed to several membrane compartments by vesicle trafficking, and eventually labels the exocytotic pathway [20,21].

We report that asymmetry in the endomembrane system in *Silvetia *zygotes developed as early as 4 h AF, and was tightly coupled to polar secretion of adhesive. Endomembrane polarity was labile and reoriented with the same time course as polar adhesive when the developmental axis was respecified. Both localized endocytosis and exocytosis likely contributed to endomembrane polarity. The physiological requirements for endomembrane polarization were investigated using pharmacological agents. The actin cytoskeleton and vesicle trafficking are needed to establish polarity in zygotes [8,15,25], and their inhibition disrupted endomembrane polarity. The results of microtubule inhibition were more surprising. Although microtubules are not required for polarity establishment [26], microtubule depolymerization also disrupted endomembrane polarization. We suggest that microtubules may help organize the endomembrane system, perhaps by interacting with actin arrays intimately involved in polarity establishment.

## Results

**Figure 1:**
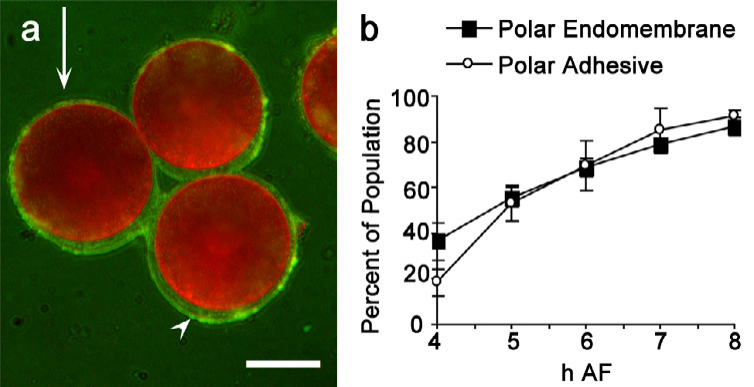
Endomembrane asymmetry in polarizing zygotes. (a) Zygotes 6 h AF were double labeled with fluorescent microspheres (green) to visualize polar adhesive (arrowhead) and FM4-64 (red) to visualize endomembrane distribution. Arrow indicates direction of orienting light vector and scale bar = 50 *μ*m. Adhesive and endomembranes accumulate at rhizoid (shaded) pole. (b) Time course of photolocalization of adhesive and endomembranes. Y axis indicates percent of zygotes with polar adhesive or polar endomembrane distribution. Over 100 zygotes were scored for each time point and the experiment was repeated three times. Bars are standard errors.

To study the spatial relationship between endomembrane distribution and development of cell polarity, zygotes were grown in unilateral light, which induced formation of a rhizoid pole on the shaded hemisphere by 4 h AF. Polarizing zygotes were doubled labeled for 30 min with FM4-64 to visualize endomembranes and fluorescent microspheres to outline adhesive. Secretion of adhesive preferentially at the rhizoid pole is the earliest visible expression of the nascent axis previously detected [8], and was used as a marker of the rhizoid pole. For the first few hours post-fertilization, endomembranes and secreted adhesive were uniformly distributed about most spherical zygotes. Preferential accumulation at the presumptive rhizoid was first detectable at 4 h AF, soon after the axis was established. Although labeled endomembranes were detected throughout the cytoplasm and adhesive was deposited over the entire surface, they became more prominent at the rhizoid pole (Fig. 1a). Most zygotes labeled either symmetrically or polarly for both parameters; cells with only polar adhesive or only polar endomembranes were less common. The percentage of zygotes with polar adhesive or polar endomembranes increased in tandem with time, and by 8 h AF over 80% of zygotes displayed rhizoid localization of both adhesive and endomembranes (Fig. 1b). There was no significant difference between endomembrane and adhesive polarization at any time point, so it was not possible to determine which polarity was established first. These data indicate that polarization of endomembranes and secretion occurred simultaneously early in development and were likely tightly coupled processes.

**Figure 2:**
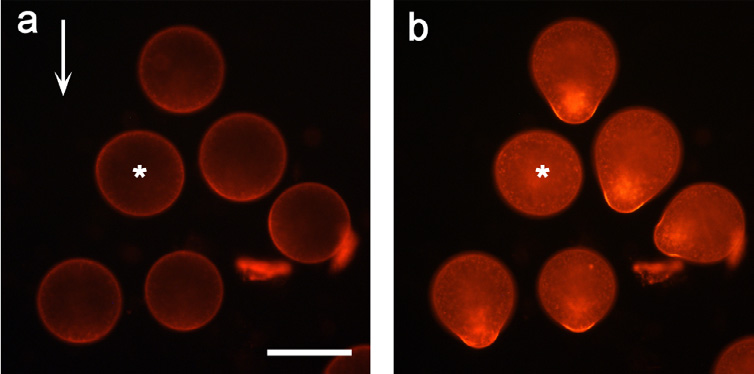
Endomembrane accumulation marks the rhizoid pole. Vital staining of a field of 6 zygotes with FM4-64 during photopolarization (a, 8 h AF) and following germination (b, 20 h AF). Each zygote germinated at the position previously marked by brightest FM4-64, except for the cell indicated by an asterisk, which had uniform labeling at 6 h and did not germinate. Arrow indicates direction of orienting light vector and scale bar = 100 *μ*m.

FM4-64 is a vital dye, allowing labeled zygotes to be followed through development. To confirm that endomembrane accumulation marked the site of future rhizoid outgrowth, 8h-old photopolarized zygotes were labeled with FM4-64 and imaged to determine the region with endomembrane accumulation (Fig. 2a). The zygotes were then placed in the dark, allowed to germinate, relabeled and imaged again at 20 h AF. Rhizoids emerged from the region of the cell that had previously labeled most brightly (Fig. 2b). One zygote (labeled with an asterisk) in the field had uniform labeling at 8 h AF and failed to germinate. Germinated zygotes labeled most intensely in the growing rhizoid where vesicles are known to accumulate. Preferential FM4-64 labeling in membranous organelles in the apical cytoplasm has been previously reported for other tip-growing cells, including *Fucus *zygotes [20-23,27].

**Figure 3:**
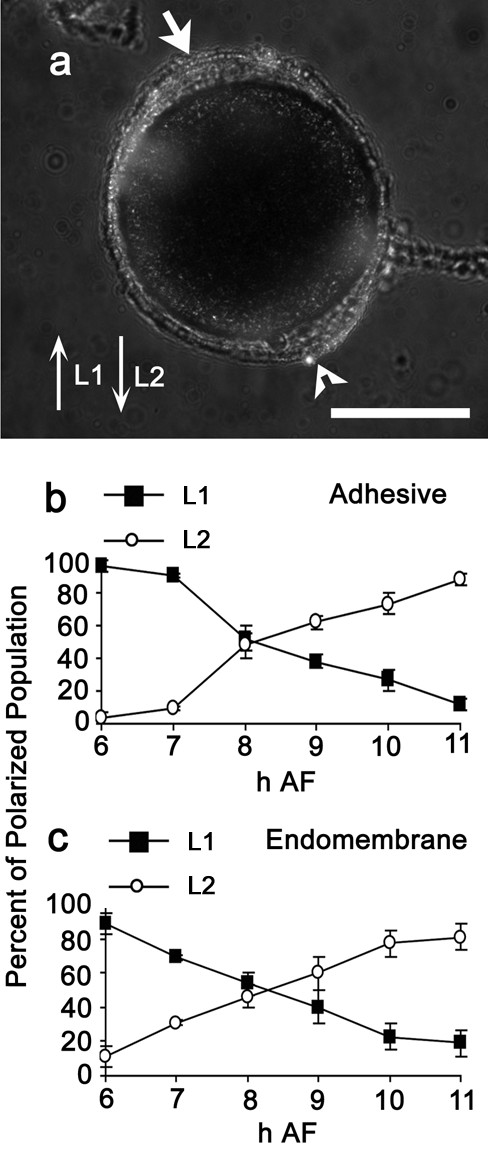
Endomembrane asymmetry can be reoriented. Zygotes were grown in unilateral light until 6 h AF (L1), at which time the light direction was reversed (L2). Zygotes were labeled for 30 min at hourly intervals beginning at 6 h AF and scored as having polar adhesive or endomembrane in accordance with L1 or L2. Zygotes with uniform labeling were not scored. (a) Adhesive deposited in accordance with L1 (arrow) was not resorbed and a second zone of polar adhesive (arrowhead) was deposited in accordance with L2. Bar = 50 *μ*m. (b) Adhesive deposition reversed following light reversal. Note that filled squares represent adhesive localization with L1 and open circles represent zygotes with two patches of polar adhesive. (c) Endomembrane localization with L1 (filled squares) decreased with time after light reversal and localization with L2 (open circles) increased. Over 100 zygotes were scored for each time point and the experiment was repeated three times. Bars are standard errors.

In fucoid zygotes the developmental axis is initially labile and can be reoriented by changing the direction of the orienting vector [28,29]. To determine whether endomembrane asymmetry can be repositioned, zygotes were exposed to two sequential light vectors from opposite directions. Zygotes were grown in grown in unilateral light (L1) until 6 h AF, at which time the light direction was reversed (L2). Adhesive and endomembranes were labeled every hour beginning at 6 h AF. Previous studies have shown that adhesive is not resorbed when the axis is respecified [8], and individual zygotes therefore bore polar adhesive deposited in accordance with L1 (arrow, Fig. 3a) and with L2 (arrowhead, Fig. 3a). The percentage of zygotes with adhesive deposited in accordance with L2 steadily increased from 7 to 11 h AF (Fig. 3b). Endomembrane asymmetry was also repositioned when the light vector was changed. In contrast to adhesive, endomembrane labeling gradually faded at the initial rhizoid pole and increased at the new pole induced by L2. Endomembrane labeling was scored as brightest in accordance with L1 or L2; zygotes with uniform labeling (5–10% of population at all time points) were not included. At the time of light reversal (6 h AF), nearly 90% of zygotes with asymmetric labeling showed polar endomembranes in accordance with L1 (Fig. 3b). By 8 h AF approximately half of the zygotes showed preferential labeling with L1 and half with L2, and by 11 h AF ~80% of zygotes had endomembranes localized in accordance with L2. The time course of endomembrane relocalization was nearly identical to that for reversal in adhesive deposition, again indicating that the two processes were coupled.

**Figure 4:**
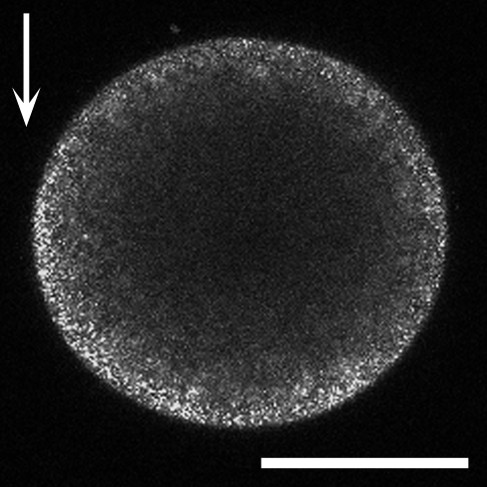
Endocytosis contributes to polar endomembrane labeling. A 6 h-old zygote was labeled for 2 min and the spatial distribution of endocytosis was immediately imaged by confocal microscopy. Image is a single optical section near the median plane demonstrating preferential FM4-64 uptake at the rhizoid pole. Arrow indicates direction of polarizing light and bar = 50 *μ*m.

To determine whether localized endocytosis contributed to the asymmetric endomembrane distribution, photopolarized zygotes were labeled for short times (1 to 30 min) with FM4-64 and immediately imaged by confocal microscopy. Within 1 min of addition of FM4-64, the plasma membrane became brightly fluorescent over the entire cellular surface. By 2 min, the dye was present in punctate structures in the cortical cytoplasm (Fig. 4). Although uptake occurred over the entire cell surface, labeling was most intense at the presumptive rhizoid pole. When 6-h old zygotes were stained for 1–5 min, 56% (n = 28) preferentially labeled at the rhizoid pole and the remainder displayed a more uniform fluorescence. With time the dye penetrated deeper into the cell, reaching the perinuclear region by 15 to 30 min. Labeling remained asymmetric throughout this time period. These data indicate that localized endocytosis contributed to asymmetric endomembrane labeling. Eventually the dye entered the Golgi and secretory pathway and labeled newly deposited membrane at the cell plate [20]. Membrane trafficking probably redistributed the dye to other internal membranes, but the specific organelles labeled were not investigated.

**Figure 5:**
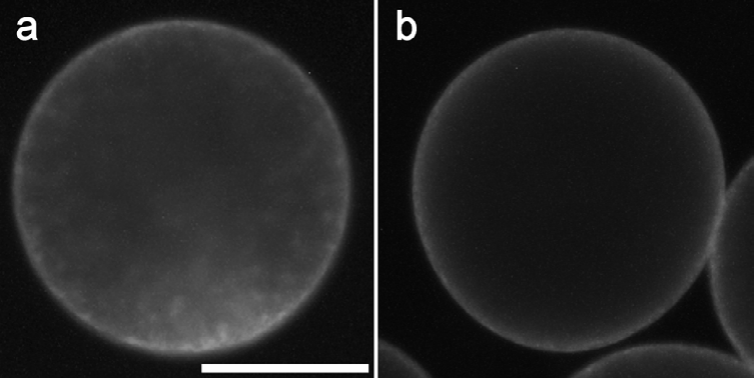
Endocytosis is inhibited by actin disruption. Zygotes 6 h AF were treated for 30 min with DMSO (a) or 30 nM Lat B (b) and then labeled with FM4-64 for 30 min in the presence of drug. Diffuse cortical fluorescence in (b) is primarily due to out-of-focus labeling of plasma membrane. Bar = 50 *μ*m.

In most cell types examined, the actin cytoskeleton facilitates endocytosis [3,9]. The role of actin in polar endocytosis was examined by treatment with 30 nM Lat B, which depolymerizes actin filaments in *S. compressa *zygotes [8,15]. 6-h old zygotes were treated with Lat B for 1 h and then labeled with FM4-64 for 30 min. In control zygotes, endomembranes were asymmetrically distributed toward the rhizoid pole and organelles deep into the cytoplasm were clearly labeled (Fig. 5a). In contrast, FM4-64 incorporated into the plasma membrane in Lat B-treated zygotes (Fig. 5b), but was not internalized to membranous organelles in any of the zygotes examined (n > 300). Since FM4-64 enters the cell exclusively by endocytosis and does not diffuse across membranes [2,24], inhibition of uptake by Lat B indicates that actin facilitates endocytosis in fucoid zygotes.

**Figure 6:**
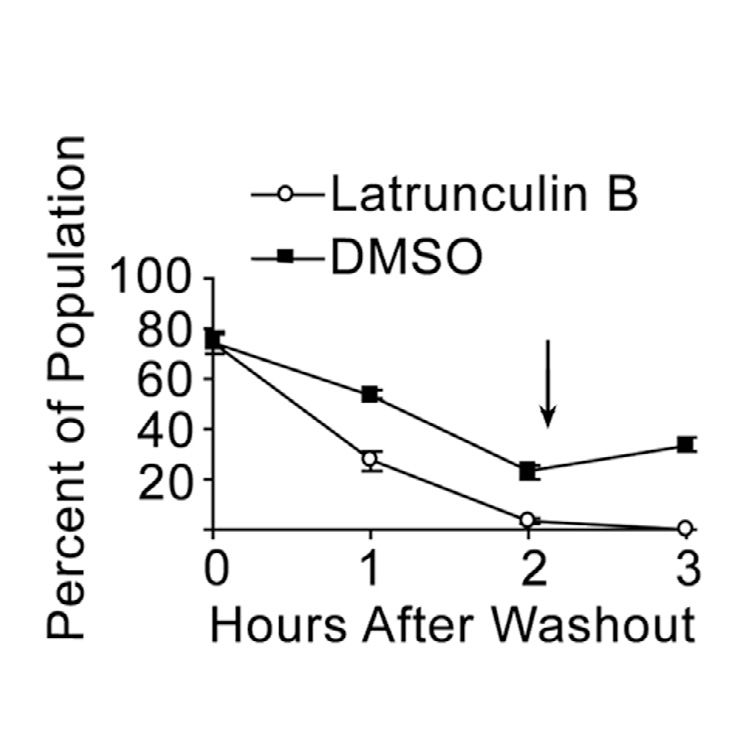
Actin disruption accelerates loss of polar endomembrane labeling. At 6.5 h AF, zygotes on coverslips in Petri dishes were labeled for 30 min followed by 3 × 5 min rinses in ASW to wash out FM4-64. Zygotes were immediately scored for initial endomembrane polarity. Either 0.06% DMSO or 30 nM Lat B was then added to a dish and endomembrane polarity was again scored 1 and 2 h later on separate coverslips. At the time indicated by the arrow (2 h after FM4-64 washout), the media were spiked with FM4-64. Y axis indicates percent of zygotes with polar endomembrane distribution. Over 100 zygotes were scored for each point and the experiment was repeated three times. Bars are standard errors.

Since Lat B prevents internalization of FM4-64, the role of actin in maintaining endomembrane asymmetry was investigated by loading the zygotes with dye prior to treatment. Polarizing zygotes (6.5 h AF) were loaded with FM4-64 for 30 min, the dye was then washed out and zygotes were immediately assayed for polar endomembrane labeling. At this time, nearly 80% of zygotes showed preferential rhizoid labeling (Fig. 6). Zygotes were then treated with either Lat B (30 nM) or DMSO. Endomembrane labeling was assayed 1 h and 2 h later. Control and treated zygotes remained labeled throughout the duration of the experiment. With time, DMSO-treated control zygotes gradually lost asymmetric labeling, probably because membrane trafficking redistributed dye to organelles that were more uniformly distributed throughout the cytoplasm [20,30]. 2 h after washout, asymmetric labeling was observed in ~23% of control zygotes. Lat B treated zygotes lost asymmetric labeling faster than controls, and after 2 h less than 4% showed clear asymmetry. These findings are consistent with actin participating in maintaining the polar endomembrane array. Fresh FM4-64 was added after the 2 h time point (arrow) to permit endocytosis anew, and labeling was again assayed 1 h later (3 h after initial washout). As expected, asymmetry increased in controls due to polar endocytosis of dye, whereas Lat B-treated zygotes did not regain asymmetry because endocytosis was blocked.

**Figure 7:**
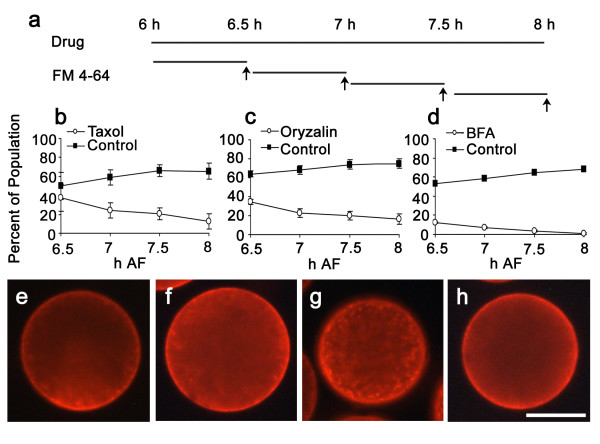
Perturbation of microtubule dynamics or membrane trafficking alters endomembrane distribution. (a) Experimental design. Zygotes on coverslips in Petri dishes were treated chronically with drug beginning at 6 h AF. At 30 min intervals thereafter a separate coverslip was labeled with FM4-64 for 30 min. Arrows at the end of the FM4-64 labeling period indicate time at which zygotes were scored for endomembrane asymmetry. (b-d) Time course of changes in endomembrane asymmetry following treatment with 5 *μ*M paclitaxel (b), 5 *μ*M oryzalin (c), or 5 *μ*g/ml BFA (d). Y axis indicates percent of zygotes with polar endomembrane distribution. Each experiment was repeated three times and over 100 zygotes were scored for each time point. Bars are standard errors. Images are zygotes treated with 0.05% DMSO (e), paclitaxel (f), oryzalin (g) or BFA (h). Bar = 50 *μ*m

The role of microtubules in organizing an asymmetric endomembrane array was examined by stabilizing microtubules with 5 *μ*M paclitaxel (taxol) or depolymerizing microtubules with 5 *μ*M oryzalin [31,32]. Neither treatment blocked dye uptake, indicating that microtubules are not required for endocytosis, so preloading zygotes with FM4-64 prior to treatment was not necessary. Zygotes were grown on coverslips in unilateral light to allow polarization of the endomembrane system and then treated with pharmacological agents at 6 h AF. Every 30 min thereafter, a separate coverslip with treated zygotes was labeled with FM4-64 and endomembrane asymmetry was scored (Fig. 7a). Because FM4-64 was not washed out, asymmetry slowly increased in DMSO-treated control zygotes. In contrast, zygotes treated with paclitaxel or oryzalin gradually lost endomembrane asymmetry (Fig. 7b,c). Labeling remained bright, but became more uniformly distributed throughout the cytoplasm with time (Fig. 7e–g). Microtubules, like filamentous actin, appear to participate in organizing the polar endomembrane array.

BFA generally inhibits small GTPases involved in membrane trafficking [9,33,34], and is thought to perturb actin-dependent endosomal cycling in plants [2]. Although the mechanism of action of BFA has not been investigated in fucoid zygotes, it has been shown to disrupt Golgi [35] and to inhibit photopolarization [8] and the accompanying local deposition of adhesive and a cell wall polysaccharide, fucoidin, in response to an orienting light vector [8,25]. The effects of BFA on the endomembrane array in polarizing zygotes were examined as described above for paclitaxel and oryzalin. Although BFA treatment has been reported to increase uptake of FM dyes in plant suspension culture cells [34], we did not observe noticeable changes in FM4-64 uptake in treated fucoid zygotes. However, BFA induced disorganization of polar endomembrane arrays within 30 min (Fig. 7d). Labeling became symmetrically distributed throughout the zygote (Fig. 7h), indicating that normal membrane trafficking is needed to maintain endomembrane polarity.

## Discussion

### Endomembranes localize to the rhizoid pole during photopolarization

Polar endomembrane arrays targeted to the elongating apex are a common feature of tip growth in fungi, algae and plants [5,9,36]. However, the mechanisms that establish and maintain endomembrane polarity are not well understood. Fucoid zygotes offer an excellent opportunity to investigate spatial and temporal aspects of the transition from symmetrical to polar endomembrane arrays. We found that that polarity can be detected early in development (4 h AF) and that the percentage of zygotes with polar endomembranes increases over the next few hours. These findings contrast with previous reports indicating that endomembrane polarity was acquired around the time of germination [19,20], and instead support a preliminary report of early polarization [6]. One possible reason for the apparent discrepancy concerns the organisms investigated. The studies reporting late endomembrane polarization were done in *Fucus *whereas early polarization was found with *Silvetia*. Although most aspects of cell polarity are thought to be identical in the two genera, adhesive deposition at the rhizoid pole occurs later in *Fucus *(6–8 h AF) than in *Silvetia *(4–6 h AF) [8]. Since we found a tight temporal correlation between adhesive and endomembrane polarizations, it seems likely that endomembrane polarization occurs 6–8 h AF in *Fucus*, several hours prior to germination.

Both local endocytosis and targeted secretion at the rhizoid pole likely contribute to the polarization of the endomembrane array. Direct observation of endocytosis following brief labeling with FM4-64 indicated that endocytosis occurs preferentially at the rhizoid pole. Endocytosis appears to be a dominant pathway for localizing the endomembrane array since FM4-64 washout, which blocks further dye uptake, caused a marked loss in asymmetry. The temporal coupling between polar adhesive deposition and endomembrane polarization is consistent with the interpretation that secretory pathway components accumulating in the rhizoid hemisphere strengthen endomembrane asymmetry. Effects of BFA treatment further support a localized secretory pathway; BFA, which blocks Golgi trafficking and secretion in *Silvetia *zygotes [8,35], caused a rapid loss of endomembrane asymmetry. It should be noted that the endo/exocytotic pathways comprise a subset of endomembranous organelles, many of which do not become localized. Physodes, chloroplast and several populations of vesicles are uniformly distributed during early development and photopolarization [30,37,38]. The observation that FM4-64 washout in untreated zygotes caused a gradual loss of asymmetric labeling (see Fig. 6) is consistent with membrane trafficking between localized and uniformly distributed compartments.

Although targeted secretion is often a late event in polarity establishment, occurring just prior to the onset of tip growth [1], we find that both the endocytotic and exocytotic limbs of the endomembrane pathway become polarized many hours before rhizoid outgrowth, while the developmental axis is still labile. Endomembrane asymmetry is detectable shortly after photolocalization of a cortical patch of actin at the rhizoid pole [15]. These findings are consistent with the cortical actin patch serving as a target site for both endocytosis and secretion [5,11]. Although the pathway responsible for targeting secretion to the actin patch is unknown, recent work in *Fucus *has identified two small GTPases that may be involved [30]. FdRac1 is in the Rho family, which controls actin assembly, and is present in zygotes throughout the period of polarization. In germinated zygotes, FdRac1 localizes in the cortex at the rhizoid tip, suggesting that it may regulate actin assembly and dynamics. The second GTPase, FdRab8, belongs to the Rab family, which is involved in vesicle trafficking. FdRab8 also localizes to the rhizoid of germinated zygotes, but its localization and function during polarization has not been investigated.

The spatial relationships between the actin patch, endomembrane distribution and secretory site are more complex. Secretion of polar adhesive is broadly spread over the rhizoid hemisphere, similar to the distribution of endomembranes, and contrasts with the more tightly focused cortical actin domain. Yet, at germination the diameter of the emerging rhizoid is similar to the actin patch diameter, and the position of the actin patch very accurately predicts the site of outgrowth [15]. The notion that the actin patch, not the endomembrane distribution, is the primary determinant of the growth site is supported by treatments with paclitaxel or oryzalin. These agents result in a muted, much less asymmetric endomembrane distribution and yet the treated zygotes form photopolarized rhizoids, albeit somewhat broad and misshapen [39]. Together, these observations suggest that secretion is initially broadly distributed about the actin patch and becomes more focused as growth ensues.

### Endocytosis requires actin

Endocytosis, as assayed by the initial stages of FM4-64 uptake, occurs over the entire cell surface, but is greatest in the rhizoid hemisphere of polarizing zygotes and at the elongating apex of germinated zygotes. We did not find preferential endocytosis in the subapical zone of growing rhizoids, as has been reported for other tip-growing cells [36]. Endocytosis continues after depolymerization or stabilization of microtubules, and after disruption of Golgi and membrane trafficking. However, endocytosis was substantially reduced following actin depolymerization by Lat B. Endocytosis mediated by actin is likely an evolutionarily conserved mechanism of uptake, since it is present across the eukaryotic lineage. Recent work indicates that actin polymerization facilitates formation of clathrin-coated endocytotic vesicles [3,9].

### Cellular requirements for endomembrane polarity

Cellular and physiological aspects of establishment and maintenance of endomembrane polarity were investigated using pharmacological agents. Since actin depolymerization by Lat B inhibited endocytosis of FM4-64, the role of actin in maintaining an asymmetric array was investigated by loading the zygotes with dye prior to treatment. Actin depolymerization accelerated the loss of endomembrane polarity. One possibility, which we do not favor, is that actin depolymerization accelerated vesicle trafficking between organelles, including those that are symmetrically distributed. This could speed redistribution of FM4-64 throughout the cytoplasm. However, in algae and plants, vesicle trafficking occurs along actin filaments and actin depolymerization would therefore be expected to slow, rather than accelerate, trafficking [40]. We instead favor the interpretation that actin structurally organizes the polar endomembrane array, as has been reported for the initial stages of root hair bulging in plants [41]. One problem with this interpretation is that actin in polarizing zygotes localizes to the nuclear surface and a cortical patch, but little is observed in the intervening cytoplasm where endomembranes accumulate. However, imaging using improved preservation techniques shows that what was once thought to be a subapical cortical actin ring in elongating rhizoids [15] is instead a branching Arp2/actin network that extends from the nucleus to the rhizoid apex [42]. In growing rhizoids the distribution of the Arp2/actin network is remarkably similar to the FM4-64 labeling pattern reported here, suggesting a causal link. If such a cytoskeletal network were present in the rhizoid hemisphere of polarizing zygotes, it could function in accumulating and stabilizing endomembranes at that site. Ongoing work using high-pressure freezing techniques will address this issue.

Perhaps the most surprising finding of the pharmacological studies was the observation that perturbation of microtubule arrays induced a loss of polarity in the endomembrane system. Zygotes photopolarize and form rhizoids following microtubule depolymerization or stabilization [26,39], which has been interpreted to mean that microtubules are not required for the polarization process. Rhizoids formed by treated zygotes are relatively fat and misshapen, indicating that microtubules play an accessory role in tip morphogenesis. Similar results have been found in tip-growing plant cells; for example, in *Arabidopsis*, root hairs are formed following microtubule perturbation, but they elongate in a wavy pattern [43,44]. It has been suggested that microtubules determine the direction of root hair tip growth by guiding the actin/endomembrane system that is directly responsible for elongation [45].

Microtubules may also help organize the endomembrane system in fucoid zygotes. Unfortunately, the spatial distribution of microtubule arrays in polarizing fucoid zygotes is at present unclear. When viewed by confocal immunofluorescence microscopy, microtubules extend radially from perinuclear centrosomes to the plasma membrane where they bend and extend further along the inner leaflet without detectable spatial asymmetry [46,47]. However, when viewed in living cells following fluorescent tubulin injection, distinct centrosomal and cortical microtubule arrays are detected and the cortical array is polarized toward the rhizoid pole [48]. Since the endomembrane system labeled with FM4-64 extends from the perinuclear region to the rhizoid cortex, the radial centrosomal microtubules are well positioned to organize the endomembrane system into a polar array targeted to the rhizoid pole. However, a role for cortical microtubules cannot be ruled out.

One intriguing possibility is that microtubules interact with actin to organize endomembranes. Microtubule-actin interactions are often mediated by plus end tracking proteins (+TIPs) that bind to the plus ends of dynamic microtubules and facilitate microtubule capture at actin rich sites in the cell cortex. Such interactions play important roles in cell polarization and spindle positioning in metazoans, yeast and perhaps in higher plants [49,50]. We have preliminary evidence that a +TIP of the EB1 family binds to the plus end of microtubules in fucoid zygotes (Sherryl Bisgrove, unpublished observation). During polarization, EB1 and its binding partners may mediate microtubule capture at the cortical actin patch (or Arp2/actin network). This would selectively stabilize microtubules at the rhizoid pole, and these microtubules may then interact with, and locally organize, endomembranes into polar arrays.

## Methods

Sexually mature receptacles of the monoecious species, *Silvetia compressa *(J. Agardh) E. Serrao, T. O. Cho, S. M. Boo et Brawley were collected near Pigeon Point Lighthouse, north of Santa Cruz, CA. Receptacles were shipped cold and stored in the dark at 4°C for up to 3 weeks. To induce the release of gametes, receptacles were potentiated by placing them in uniform light (100 *μ*mol•m^-2^•s^-1^) at 16°C in artificial sea water (ASW: 0.45 M NaCl, 10 mM KCl, 9 mM CaCl_2_•2H_2_O, 30 mM MgCl_2_•6H_2_O, 16 mM MgSO_4_, and 40 *μ*g/ml chloramphenicol, buffered to pH 8.2 with 10 mM Tris base) until they were observed to accumulate small gas bubbles on their surfaces (4 h to overnight). Potentiated receptacles were rinsed with ASW and transferred to the dark for 30 min, during which time gamete release occurred. The time of fertilization was considered to be 15 min after transfer to the dark. Zygotes were obtained by filtration through nylon mesh, rinsed 2 to 3 times with ASW and cultured in ASW at 16°C either in unilateral light (100 *μ*mol•m^-2^•s^-1^) or in the dark.

Routinely, polarizing zygotes (4–8 h AF) grown on coverslips were labeled with 5 *μ*M FM4-64 (Molecular Probes Inc., Eugene OR; stock = 20 mM in DMSO) for 30 min and observed by conventional epifluorescence microscopy using a 577.5–632.5 nm band pass emission filter (Chroma Technologies, Brattleboro, VT). Images were captured with a Cool-Snap digital camera (Roper Scientific Photometrics, Tucson AZ). Confocal images were collected on a LSM510 (Carl Zeiss, Inc., Thornwood, NY) using a 685 nm short pass emission filter.

Treatments with pharmacological agents were begun at different times during polarization and were continuous in most experiments. Actin filaments were depolymerized with 30 nM latrunculin B (Lat B) [8], and microtubules were stabilized with 5 *μ*M paclitaxel (taxol) or destabilized with 5 *μ*M oryzalin [31]. Membrane cycling was disrupted with 5 *μ*g/ml brefeldin A (BFA) [33]. Stock solutions were made as follows: Lat B (Calbiochem, La Jolla, CA), 50 *μ*M in DMSO; paclitaxel (Sigma-Aldrich, St. Louis, MO), 10 mM in DMSO; oryzalin, 10 mM in DMSO; BFA (Sigma-Aldrich, St. Louis, MO), 2.5 mg/ml in ethanol. Appropriate solvent controls were included in all experiments and were found to have no effect on development. Each experiment was done in triplicate and over 100 zygotes were scored for each point on a graph.

## Abbreviations

AF, after fertilization; ASW, artificial seawater; BFA, brefeldin A; DMSO, dimethyl sulfoxide ; FM4 - 64, N - (3 - triethylammoniumpropyl) - 4 - (6 - (4 -(diethylamino)phenyl)hexatrienyl)pyridinium dibromide; LatB, latrunculin B; +TIP, plus end tracking protein

## Authors' contributions

Rhett Hadley and Whitney E. Hable conducted the research and Darryl L. Kropf supervised the entire project.
